# Effect of Different Routes of Vaccination against *Aeromonas salmonicida* on Rearing Indicators and Survival after an Experimental Challenge of Pikeperch (*Sander lucioperca*) in Controlled Rearing

**DOI:** 10.3390/vaccines8030476

**Published:** 2020-08-26

**Authors:** Patrycja Schulz, Elżbieta Terech-Majewska, Andrzej Krzysztof Siwicki, Barbara Kazuń, Krystyna Demska-Zakęś, Maciej Rożyński, Zdzisław Zakęś

**Affiliations:** 1 Department of Microbiology and Clinical Immunology, Faculty of Veterinary Medicine, University of Warmia and Mazury, 10-719 Olsztyn, Poland; siwicki@uwm.edu.pl; 2 Department of Epizootiology, Faculty of Veterinary Medicine, University of Warmia and Mazury, 10-719 Olsztyn, Poland; etam@uwm.edu.pl; 3 Department of Fish Pathology and Immunology, Stanislaw Sakowicz Inland Fisheries Institute, 10-719 Olsztyn, Poland; b.kazun@infish.com.pl; 4 Department of Ichthyology, Faculty of Environmental Sciences, University of Warmia and Mazury, 10-719 Olsztyn, Poland; krysiadz@uwm.edu.pl; 5 Department of Aquaculture, The Stanislaw Sakowicz Inland Fisheries Institute, 10-719 Olsztyn, Poland; m.rozynski@infish.com.pl (M.R.); z.zakes@infish.com.pl (Z.Z.)

**Keywords:** vaccines, autovaccines, specific immunity, furunculosis, immune response

## Abstract

Bacterial diseases are a significant problem in the controlled rearing of fish. Furunculosis (*Aeromonas* sp.), flavobacteriosis (*Flavobacterium* sp.), and pseudomonadosis (*Pseudomonas* sp.) are currently the most frequently identified diseases in recirculating aquaculture systems of various fish species. Such a situation is also observed in pikeperch rearing. Due to the emerging difficulties of effective prophylaxis using commercial vaccines, interest in the use of autovaccinations is increasing, not only in ichthyopathology but also in other veterinary fields. Our research aimed to assess the effect of the vaccination method on the overall condition of the fish and survival after the experimental infection with *Aeromonas salmonicida*. Pikeperch were vaccinated by (1) bath, (2) a single i.p. injection, or (3) feed. The fish were measured and weighed on day 0 and after 28 and 56 days of the experiment. Specific growth rate, daily growth rate, condition factor, and feed conversion ratio were calculated. On days 7, 14, 21, and 28 of the experiment, ceruloplasmin and lysozyme levels were rated. In addition, a challenge test was performed. The obtained results showed that the method of vaccination is important and affects the growth of fish, the overall condition of fish, and survival after experimental infection.

## 1. Introduction

The continuous increase of fish production by increasing the density of animals, along with increasing the pollution of the environment, significantly contributes to the development of many infectious diseases. Fish are very often bred in monocultures, which disturbs the natural biological balance in the aquatic environment. The appearance of a specific pathogen in a facility where fish susceptible to this factor are bred creates ideal conditions for the development of the disease, which can progress very quickly. The further development of freshwater aquaculture requires the diversification of its production through the introduction of new species of fish. Over the past two decades, attempts have been made to develop a culture of common perch (*Perca fluviatilis*) and pikeperch (*Sander lucioperca*). Both species are valuable commercial fish and have an acceptable growth rate to market size in conditions of intense culture. Pikeperch is also a valuable recreational species because currently most pikeperch come from open waters (lakes, rivers, and ponds), and relatively few are produced under intense conditions and in closed circuits. As a consequence, their availability on the market varies strongly, as does their market price and meat quality. The market exists for all life stages of pikeperch [1].

Almost every year, freshwater fish health disorders are noted on many farms. Despite an improvement in the aquaculture sector, it is estimated that as much as 10% of all aquatic aquaculture animals die of infectious diseases, representing losses of more than USD 10 billion per year globally [2]. Diseases can be caused by various factors; however, the most important among bacterial infections are those caused by motile *Aeromonas*. The genus *Aeromonas* comprises a collection of Gram-negative bacteria that are widespread in the aquatic environment. Many *Aeromonas* species are opportunistic bacteria. They can also have symbiotic relationships with animal hosts or be commensals. *A. salmonicida* has a broad host range, affecting salmonid species and a variety of non-salmonid fish. Furunculosis caused by *Aeromonas salmonicida* subsp. *salmonicida* (*A. salmonicida*) is a ubiquitous disease that affects aquaculture worldwide and is characterized by high mortality and morbidity. It is a complex disease that can have a different clinical picture and course depending on the health, age, and species of fish, as well as environmental conditions [3]. Outbreaks are caused by stressors such as sudden water temperature changes, handling, excessive stocking density, and poor water quality. In the acute form, the disease quickly leads to sepsis [4]. An acute form causes sudden mass death with no obvious clinical symptoms, except for darker skin pigmentation, lethargy, and loss of appetite. Death occurs within 2–3 days after infection [3]. In the subchronic and chronic forms of the disease, fish can show symptoms such as lethargy, lack of appetite, pale gills, and darkening of the skin, necrotic skin and muscle defects, and fin shredding. These are not pathognomonic symptoms and are also reported for other bacterial diseases. Anatomopathological changes include renal necrotic foci, splenomegaly, and peritoneal hyperemia. Hemorrhages in inner organs and at the base of the fins can also be observed [5]. A carrier state may become established in surviving fish after an infection.

In case of disease, even the use of an antibiotic does not always improve fish health, because these drugs are usually given in feed, and sick fish stop eating [6]. Choosing the right antibiotics for a bacterial disease is time-consuming. Therefore, the sustainable development of aquaculture is based on the prevention of diseases [7]. Vaccinations play an important role in commercial large-scale fish farming. They stimulate the immune system to help relieve the symptoms of the disease and are increasingly important in controlling infectious diseases. Different vaccination methods have been applied to protect fish against the harmful effects of various pathogens. The methods used include vaccination by mucosal surfaces (immersion or oral) or injection. Each of these methods has advantages and disadvantages. Most vaccines in aquaculture are injected. However, this method is the most labor-intensive [8]. The advantages of injection vaccines are that the required volume of the vaccine is relatively low, and each fish is vaccinated with the appropriate dose. Unfortunately, this method requires the fish to have reached a certain size, which makes it difficult to vaccinate the fry [9]. Immersion vaccination gives the possibility of mass vaccination while exposing the fish to moderate stress. Unfortunately, it is usually associated with the need to have large volumes of a vaccine. Oral vaccination is an ideal method of administering a vaccine to fish because it does not cause increased stress in fish, especially when administered by feed.

Commercially available vaccines are often polyvalent vaccines with oil adjuvants. The use of such vaccines is often accompanied by side effects. Due to the emerging difficulties of effective prophylaxis using commercial vaccines, interest in the use of autovaccinations is increasing, not only in ichthyopathology but also in other veterinary fields.

Our research aimed to check the effectiveness of various methods of vaccination using an autogenous vaccine against *A. salmonicida* and the impact of various administration routes on breeding parameters and survival rate of juvenile pikeperch after experimental challenge

## 2. Materials and Methods

The experiment was carried out in conformity with Animal Protection Law and the recommendations of the Animal Ethics Committee of the University of Warmia and Mazury in Olsztyn 24/2011-2016.

### 2.1. Animals and Rearing Conditions

The experimental material was juvenile pikeperch (age 0+) obtained through artificial reproduction conducted at the Inland Fisheries Institute in Olsztyn, Poland. After six months of rearing, 288 juvenile pikeperch individuals with an initial mean body weight of approximately 35 g and initial mean body length of approximately 14 cm were stocked into 12 rearing tanks (24 fish per tank) with a volume of 0.2 m^3^ each in separate recirculating systems. The initial stocking density was 4.0–4.2 kg m^−3^. Water temperature; contents of oxygen, total ammonia nitrogen (TAN = NH_3_-N + NH_4+_-N), and nitrites (NO_2_-N); and pH at the outflows of the rearing tanks were as follows: 21.8 ± 0.4 °C, 7.14 ± 0.33 mg O_2_ L^−1^, 0.05 ± 0.01 mg TAN L^−1^, 0.07 ± 0.02 NO_2_-N L^−1^, and pH 7.9–8.1. The tank water flow rate was 4 L min^−1^. A photoperiod of 24L:0D was applied (light intensity at the surface of the tanks was 30–45 lx).

For the challenge test, a separate group of 96 fish was used (4 fish groups × 3 tanks; 8 fish/tank).

### 2.2. Feed

The fish were fed commercial trout feed (Nutra T-T MP, Skretting, Sjøhagen, Norway). According to manufacturer data, the feed contained 50 g crude protein 100 g^−1^ feed of wet weight (w.w.), 20 g crude fat 100 g^−1^ w.w., 9.5 g carbohydrates 100 g^−1^ w.w., 11 g crude ash 100 g^−1^ w.w., and digestible energy of 19.9 MJ kg^−1^. Feed was delivered by automatic band feeders (4305 FIAP; Fish Technic Gmbh, Ursensollen, Germany) for 18 h per day (10:00 to 04:00). The daily feed ration was 1.5% of stocking biomass.

### 2.3. Vaccine

A pathogenic *A. salmonicida* strain isolated from a clinical field case was chosen for the development of the vaccine, which was prepared after a 48-h incubation on TSA (Triptic Soya Agar, Oxoid) at 25 °C. The bacterial suspension was then prepared in phosphate-buffered saline (PBS, physiological saline) at a concentration of 8 MF (McFarland scale). Formalin (37.8% formaldehyde solution, Polchem, Poland) was used to kill the bacteria in a concentration of 8 mL L^−1^ for 24–48 h. The efficacy of the procedure was confirmed by control culture on TSA medium for 24–48 h at 25 °C. The fish were vaccinated in three procedures: by bath, by immersion, and by feed.

For the bath vaccine, a basic solution of the vaccine was used for the treatment, which was diluted in a ratio of 1:500 (80 mL of solution was dissolved in a 40-L tank). The fish were immersed for 0.5 h. At that time, the water in the tank was additionally aerated. After the bath, the fish were returned to the recirculating aquaculture systems (RAS) tanks.

For the injection vaccine, fish were given a single intraperitoneal injection of a suspension of killed bacteria in PBS (0.2 mL/fish at a concentration of 0.5 MF). Before the injection, the fish were placed in a container with an anesthetic solution of Propiscin (etomidate; IFI Olsztyn, Poland) at 1 mL L^−1^ [10]. Then, each fish was placed on a flat surface, placed on its right side, and held in the middle of its body. The vaccine was injected between the pelvic and anal fin below the lateral line on the left side of the fish’s body. The injection was performed with a tuberculin syringe with a needle of 0.5 × 16 mm.

For the feed vaccine, a suspension of killed bacteria was used to prepare the vaccine in the feed. After being washed in PBS twice, this suspension was again diluted and added to the feed in the proportion of 10 mL of the cell suspension to 200 g of feed. The feed was then vacuum sealed with a vacuum pump (AGA Labor, Lublin, Poland).

### 2.4. Experimental Procedure

Fish were randomly divided into four experimental groups (*n* = 72): group C, not vaccinated; group I, a group with a single intraperitoneal injection of autovaccine; group B, a group bathed for 0.5 h in autovaccine solution; and group F, a group fed with commercial feed enriched with the vaccine for a period of 7 days (day 1 to day 7) and then fed commercial feed for the next 7 weeks.

The fish were reared for 8 weeks to carry out the rearing and immunological assays.

Eight weeks after vaccination, a batch of fish to carry out the experimental infection, i.e., the challenge test (CHT), was isolated from all experimental groups. For this purpose, the fish were transported in oxygen bags to the Experimental Animals Laboratory of the Faculty of Veterinary Medicine, University of Warmia and Mazury in Olsztyn. After the acclimation period to the new environmental conditions (14 days of staying in the second facility), the CHT was carried out.

### 2.5. Rearing Parameters

On the initial day (d0) and after 28 days (d28) and 56 days (d56) of the experiment, the fish were weighed (W ± 0.01 g) and measured (body length (BL ± 0.1 cm)). The fish were anesthetized in a 1.0 mL L^−1^ Propiscin (IFI Olsztyn) solution. The data collected served for calculating the following parameters: specific growth rate (SGR) in percent per day, calculated as SGR = 100 × ((lnWf − lnWi) × T^−1^); daily growth rate (DGR) in grams per day, calculated as DGR = (Wf − Wi) × T^−1^; condition factor (CF), calculated as CF = 100 × (W × BL^−3^); and feed conversion ratio (FCR), calculated as FCR = TFI × (FB − IB)^−1^. Here, Wf and Wi are the final and initial body weights (g), T is time of rearing (days), BL is body length (cm), FB and IB are the final and initial absolute weights (g fish), and TFI is total feed intake (g).

### 2.6. Immunological Assays

Nine fish from each group (three from every tank) were sampled on days 7, 14, 21, and 28 of the experiment for the analyses of the influence of different routes of vaccination on immunity parameters. Blood samples were collected from a caudal vein for blood serum and stored at −80 °C until analysis.

#### 2.6.1. Ceruloplasmin Level

Ceruloplasmin activity in the serum was determined according to Siwicki and Anderson [11]. Optical density was read immediately at 540 nm. To calculate mean OD values, triplicate determinations were averaged.

#### 2.6.2. Lysozyme Activity

The turbidimetric assay was carried out according to Siwicki and Anderson [11] to determine serum lysozyme activity. The assay is based upon the ability of lysozyme to lyse the Gram-positive bacterium *Micrococcus lysodeikticus* (Sigma-Aldrich, Saint Louis, MS, USA), which is obtained freeze-dried. A solution of *Micrococcus lysodeikticus* in sodium phosphate buffer was mixed with plasma and incubated at 25 °C. The absorbance was measured before and after 15 min of incubation in sterile plastic tubes at 450 nm. The standard was hen egg white lysozyme (Sigma-Aldrich, Saint Louis, MS, USA). Mean OD values were calculated.

### 2.7. Histological Analysis of Livers

After the conclusion of growth phase and before the challenge (d56), the livers from eight individuals from each group (23° individuals from each tank) were excised and weighed (LW ± 0.001 g) to determine the hepatosomatic index (HSI), given in percent and calculated as HSI = 100 × (LW × W^−1^), where LW is liver weight (g) and BW is fish body weight (g). Then, samples of the livers were collected for histological analyses. After the samples were fixed in Bouin solution, they were dehydrated in ethanol and xylene, embedded in paraffin blocks, and then cut into 5-μm sections with a rotary microtome (Leica, Bensheim, Germany). The sections were affixed to degreased glass slides, dried, and then stained with hematoxylin and eosin. These preparations were analyzed under a light microscope (Nikon Eclipse E600, Tokyo, Japan). MultiScanBase v. 8.08 (Computer Scanning System Ltd., Warsaw, Poland) was used with each preparation from the different specimens to measure the diameter of 50 hepatocytes (HD) and their nuclei (ND) (± 0.01 μm), and then the nucleocytoplasmic index was calculated (INCP = HD × HD^−1^).

### 2.8. Hematological and Biochemical Analysis

After the conclusion of growth phase and before the challenge (d56), about 1 mL of blood was drawn directly from the caudal vein of each individual (*n* = 8) using a heparinized syringe (Smiths Medical International ASD, Inc., St. Paul, MS, USA). The fish were anesthetized in a 1.0 mL L^−1^ Propiscin solution. The blood samples were used to determine the hematological indexes of white blood cells (WBC), red blood cells (RBC), hemoglobin (HGB), hematocrit (HCT), and platelet count (PLT) and to calculate the values of the red blood cell indexes of mean corpuscular volume (MCV), mean corpuscular hemoglobin (MCH), and mean corpuscular hemoglobin concentration (MCHC). After centrifuging the samples at 1500× *g* for 3 min (Fresco 17, Thermo Scientific, Waltham, MA, USA), the following chemical indexes were determined in the material obtained: creatinine (CREA), total protein (TP), total bilirubin (BIL-T), alanine aminotransferase (ALT), alkaline phosphatase (ALP), aspartate aminotransferase (AST), albumin (ALB), globulin (GLB), glucose (GLU), calcium (Ca), magnesium (Mg), chloride (Cl), lactate (LACT), and ammonia (NH3). The hematological measurements were performed with a BC−2800 VET semiautomatic hematology analyzer (Mindray, Shenzhen, China), while the biochemical measurements were performed with a BS-120 chemistry analyzer (Mindray, Shenzhen, China).

### 2.9. Challenge Protocol

For the challenge test, 96 fish were used (4 fish groups × 3 tanks; 8 fish per tank).The fish were infected by intraperitoneal injection 8 weeks after vaccination with a suspension of live *A. salmonicida* bacteria in a concentration of 1 × 10^7^ CFU mL^−1^, 0.2 mL per fish.

The fish were observed three times a day for changes in behavior and clinical condition. Over a period of 2 weeks, mortalities in individual groups were counted to assess the cumulative survival rate.

### 2.10. Statistical Analysis

#### 2.10.1. Growth Data, HSI, Survival, Hematology, and Biochemistry

Statistical analysis was carried out in the Statistica 12 program (StatSoft, Inc., Tulsa, OK, USA). Before proceeding with the correct part of the analysis, the data were tested for the occurrence of normal distribution (Shapiro–Wilk test) and uniformity of variance (Levene test). The proper part of the statistical analysis was performed using the Kruskal–Wallis ANOVA test. In the case of statistically significant differences, further statistical analysis was performed through multiple comparisons. All calculations were determined to be significant at *p* ≤ 0.05.

#### 2.10.2. Immunological Assays

Mean values and standard deviations of the experiments were used for comparisons among groups. The Student’s t-test was used to determine the significant difference in immunological parameters between each group against the control. All calculations were determined to be significant at *p* ≤ 0.05. Data are reported as means ± SD.

## 3. Results

The results of the rearing indicators are summarized in Table 1. Vaccination performed with different methods did not significantly affect DGR, SGR, CF, and FCR. Significant differences in BL were noted after 28 and 56 days. The lowest BL rates were recorded in the intraperitoneally vaccinated group, in two measurement periods (after 28 and 56 days). The highest BL value was recorded in the group subjected to vaccination in a bath after 28 days. However, after 56 days, the highest BL was demonstrated in the group where the vaccine was administered with feed. The lowest BW value was confirmed in fish in the intraperitoneally vaccinated group. At the same time, the highest BW was recorded in the group bathed in the autovaccine solution.

During the experiment, pikeperch mortality only occurred in the intraperitoneally vaccinated group and only in the first 28 days (d0–d28) (Table 1).

There were no significant differences in the studied hematological parameters of pikeperch between the experimental groups, except for mean corpuscular volume (MCV) (Table 2)

The biochemical parameters of pikeperch after vaccination against *A. salmonicida* showed statistically significant differences only at the total bilirubin level in the bath-vaccinated group compared to the control group (Table 3).

Morphological and histological indicators of pikeperch liver did not differ statistically in any of the vaccinated groups compared to the control group (Table 4).

**Figure 1:**
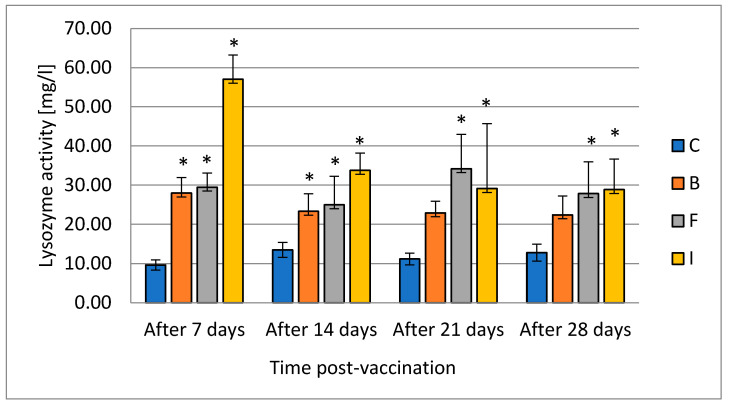
The effects of various routes of vaccination against *A. salmonicida* on lysozyme activity in pikeperch serum (mean value ± SD, *n* = 9, * statistical significance *p* ≤ 0.05).

Lysozyme activity in pikeperch serum in the bath group was increased for 14 days. In the groups where the vaccine was administered with feed and in an injection, an increase in lysozyme activity occurred during the whole experiment. The highest lysozyme activity occurred in the intraperitoneally vaccinated group 7 days after vaccination (Figure 1).

**Figure 2:**
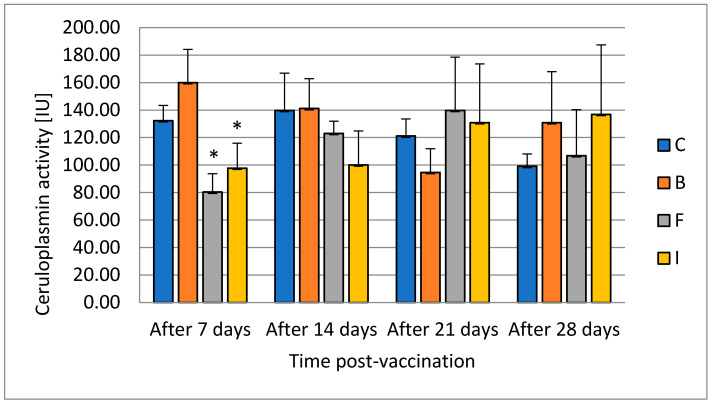
The effects of various routes of vaccination against *A. salmonicida* on ceruloplasmin activity in pikeperch serum (mean value ± SD, *n* = 9, * statistical significance *p* ≤ 0.05).

Ceruloplasmin level was lowered during the first sampling 7 days after vaccination in feed and injection (Figure 2). There was no change in this parameter in the bath-vaccinated group compared with the control.

**Figure 3:**
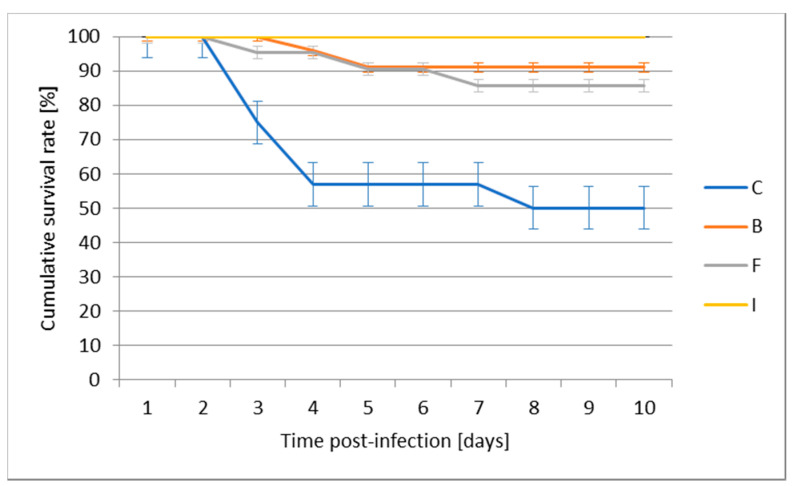
The effect of various routes of vaccination against *A. salmonicida* on the post-challenge survival rate of juvenile pikeperch.

Application of the vaccine by intraperitoneal injection before the experimental infection with *A. salmonicida* allowed a survival rate of 100% to be obtained and turned out to be the most effective protective vaccination when compared to infected control, where the survival rate was 50%. Feed vaccination provided the weakest protection against experimental infection, reaching 87% post-challenge survival rate; bath vaccination performed slightly better, with a survival rate of 91% (Figure 3).

## 4. Discussion

One of the main threats to aquaculture is economic losses resulting from outbreaks of infectious diseases, as such outbreaks cause high mortality in farmed fish and commercial aquaculture systems. Literature suggests that bacterial pathogens are responsible for almost 55% of disease outbreaks in fish farms [12]. In contrast to the treatment of humans or other animals, only few drugs are available for fish diseases. It is particularly important to be cautious when using antibiotics, which last longest in the aquatic environment, migrate through the trophic chain, and potentially produce antibiotic resistance in sediments. An additional challenge in closed systems is the protection of biofilters [13]. Therefore, the control of diseases in aquaculture and fish farms is based on the combination of good management practices and the use of several approved and commercially available drugs and vaccines [14]. Immunoprophylaxis is one of the most effective ways of preventing and controlling bacterial diseases. Its task is to increase both nonspecific and specific resistance of fish to pathogens [15,16]. The effectiveness of specific immunoprophylaxis depends on many factors. It is closely related to the environmental conditions, technology, biology and physiological needs of the species, age of fish, the maturity of the immune system, and adequate nutrition.

Vaccination for disease prevention is routinely used in fish aquaculture, especially for Atlantic salmon (*Salmo salar*); however, for many other fish species, the use of vaccination is limited due to the lack of vaccines or the high costs. Most vaccines are used in the USA, where about 30 commercial vaccines have been registered. There are 19 in Canada and 13 in Japan. Nineteen pharmaceutical companies around the world sell fish vaccines [17]. The development of a specific fish disease depends largely on the climatic conditions in the zone, region, or country. This means that different health problems occur in fish farmed in Europe compared to those farmed in Asia or America. The number of commercial vaccines available in many countries, including the Polish market, is very limited. Currently, the only registered and available vaccines in Poland are vaccines against yersiniosis and furunculosis, but they are intended for salmonids. That is why autovaccinations may be a good alternative: they are prepared for the needs of a specific fish farming facility based on bacterial strains isolated from that facility. The satisfactory effectiveness of autovaccinations is documented by many experimental studies conducted in many countries around the world [6,18].

There are three main routes of vaccine administration: immersion, injection, and oral. Each of them has different positives and negatives. Immersion vaccines are effective for many bacterial pathogens. They are cheap and easy to administer to small fish [19]. From a practical point of view, it is also the easiest method to adapt to various facilities with diverse technological levels. The immersion vaccination method gives the possibility of mass vaccination, where the fish are exposed to moderate stress. Unfortunately, it is usually associated with the need to have large volumes of a vaccine. The results obtained in this study indicate that the safest method of vaccination, taking into account the highest BL and BW rates in the period up to the 28th day, is the vaccination in a bath. There was no mortality in this experimental group during the whole period of rearing. This method is generally recognized as the safest for salmonids up to 20 g body weight. In this group of fish, immersion is most effective if we vaccinate small fish 4–10 g and the water temperature is above 10 °C [20].

Although blood parameters are a valuable indicator of the health status of aquatic organisms, they can be quite difficult to interpret due to the influence of internal and external factors. Fish hematological parameters are closely related to environmental and biological factors. Physiological changes can be a consequence of stress in response to changes in nutrition, water quality, and disease. Other factors, such as behavior, environment, and climate, can also influence hematology [21,22,23]. Variability in hematological parameters such as RBC size and volume can also be verified for the same species in different niches, inducing physiological adaptation [24]. In the hematological parameters we examined, the only parameter showing differences was MCV between the bathing group and the injection group. However, neither group differed from the control group. Smith [25] states that RBC size varies inversely to the metabolic activity of animals. Szarski [26] states that the size of red blood cells has an adaptive value; therefore, any adaptive increase in the metabolic rate would tend to decrease the size of the cells. Our results indicate an increase in metabolic activity after injection of the vaccine and a decrease in it after the bath vaccine. Changes in blood or plasma bilirubin level are often used for diagnosing liver problems in humans. Bilirubin level is considered as an indicator of liver function that assesses the ability of hepatocytes to absorb unconjugated bilirubin in the blood, make it soluble in water, and excrete the bilirubin into the bile where it is broken down by bacteria in the gut. Low bilirubin levels usually do not cause any symptoms [27]. The total amount of bilirubin in fish blood is a similar value to that in humans and other vertebrates. Depending on the fish species, it ranges from traces to several mg/dL [28]. Significantly reduced total bilirubin levels were recorded in the bath group when compared with the control, but this group did not differ from other experimental groups. None of the parameters we measured indicated liver problems. While we usually think of bilirubin in terms of its diagnostic utility, bilirubin is actually an antioxidant, which is its main physiological function. No significant difference was observed in any other biochemical parameter. After bath vaccination, the survival rate after experimental infection was 91%, which was the second best after injection.

The most effective method of vaccine administration is intraperitoneal injection. This is a particularly popular method of applying vaccines to Atlantic salmon (*Salmo salar*). The advantages of injection are that the required volume of the vaccine is relatively low and each fish is vaccinated with the appropriate dose. Unfortunately, it requires the right size and weight of the fish, due to the development of acquired immunity mechanisms and immunological memory. This is a considerable difficulty, as it limits the possibility of vaccinating the fry [19]. This type of prophylaxis is arduous, expensive, stressful for fish, and requires a minimum fish weight of 20 g [20]. In our studies, fish with average BW from 34.22 to 34.58 g were subjected to vaccination. Pikeperch with these growth rates are considered to be immunocompetent. The lowest BL and BW rates after 28 and 56 days in the injection vaccinated group may indicate the harmfulness of this method for pikeperch, but the use of commercial vaccines has also been found to cause lower feed intake and lower long term growth in other fish species [29,30,31]. In the hematological parameters of this group, the only parameter showing differences compared to the bath group was significantly reduced MCV, but this did not differ from control group. Throughout the entire breeding cycle, fish mortality occurred only in this group and only up to day 28 of the experiment, which could have been caused by stress resulting from manipulations necessary to perform the injection. However, its effectiveness can be demonstrated by a 100% survival rate after experimental infection.

One of the oldest methods of applying fish vaccines is the oral route, with the vaccine applied in feed. It was first described by Duff in the early 1940s [32]. The vaccine contained a killed *Aeromonas salmonicida* (*A. salmonicida*) strain. Currently, this method is being reintroduced. The limited number of commercial oral vaccines approved for use in humans and animals clearly indicates that the development of effective and safe vaccines for this route of administration is a challenge.

Administration of the “per os” vaccine may be even more effective if we have an appropriate granulate containing a vaccine antigen. Oral vaccination with an antigen in the feed is an ideal method of administering a vaccine to fish of almost all ages [15]. It is safe and does not cause increased stress to fish. Most often, this method is used in conjunction with injectable or immersion vaccines, in integrated prevention programs. However, if the method is used alone, it has moderate to low effectiveness [19]. Results obtained in this study show that this method is safe for juvenile pikeperch in the assessed period of rearing, in which no mortality occurred in this group. In spite of this, the lowest survival rate (87%) after experimental infection in this group confirms that its effectiveness may be limited. This is probably partly due to the breakdown of the antigen under gastric conditions but may also be due to high intestinal tolerance [33]. A summary of experimental approaches to developing oral vaccines against bacterial pathogens using non-encapsulated antigens (review in [31]) showed a survival of 100% (depending on the pathogen, time of vaccination, and fish species) which indicates that our 87% survival rate after experimental infection can be considered a good result.

Fish, due to their evolutionary history, mostly depend on nonspecific/innate immunity; the fish immune system is also less developed. The level and activity of serum lysozyme is considered to be an important indicator of the innate immunity of fish. Lysozyme has both antibacterial and antiviral activity [34]. Through its dual action as a lytic enzyme and a small cationic protein, it damages or kills bacteria by lysing cell wall peptidoglycan, destroying bacterial membranes, and activating autolytic enzymes in the bacterial cell wall. Lysozyme is secreted by the submucosal glands, neutrophils, and macrophages. The activity of lysozyme is a function of both pH and ionic strength [35]. In addition to its antibacterial effect, it promotes phagocytosis through direct activation of leukocytes and macrophages or indirectly through opsonic action. The enzyme can also damage structures containing muramic acid and hydrolyze glycolic chitin; it has a limited degrading effect on chitin, which is a major component of fungal cell walls and exoskeletons of some invertebrates. Lysozyme in fish, like in mammals, occurs mainly in neutrophils, monocytes, and in small amounts in macrophages. In fish, lysozyme, which is released by leukocytes, is especially important because fish live in an aquatic environment rich in pathogens. Fish lysozyme is found mainly in the head kidney, which is rich in leukocytes, and in places where the risk of bacterial invasion is very high, such as gills, skin, digestive tract, and eggs, which underlines the role of lysozyme. The close association of lysozyme with cells of the immune system may indicate that this enzyme contributes to defense against infectious diseases. Lysozyme has also been established as a resistance marker in the selective breeding of fish for resistance to bacterial diseases. [36]. Different data show different impacts of bacterial LPS contained in the vaccine on the lysozyme level in fish. It was noted that bacterial LPS can either increase or decrease this parameter [36]. Jiang et al. [37] vaccinated common carp with live-attenuated and formalin-killed *Aeromonas hydrophila*. The changes in lysozyme activities in that study showed no significant difference between the vaccinated group and the control, but lysozyme activity in each group was gradually decreased with the extension of time. On the contrary, hybrid surubim (*Pseudoplatystoma corruscans* × *P. fasciatum*) immunized against motile *Aeromonas hydrophila* septicemia showed an increase of lysozyme activity in groups vaccinated intraperitoneally and by immersion [38]. Our results indicate stimulation of lysozyme production in all vaccinated groups, but the increased level occurred for the longest in the groups where vaccination was performed by injection and by the feed. Results of Koskela et al. [39] concerning the effect of intraperitoneal immunization with two commercially available vaccines on European whitefish (*Coregonus lavaretus*) showed a long-term increase in the activity of lysozyme up to 49th day of the experiment, which is in line with results obtained in this study. The increase in enzyme levels may reflect changes in the white blood cell population during the development of the immune response. Thus, vaccination that causes changes in leukocyte counts may affect the concentration of lysozyme, and the assessment of lysozyme may be diagnostic in determining fish disease status [38].

Ceruloplasmin is an element of the innate humoral response. It inhibits the growth of bacteria by depriving them of access to copper ions. It is an acute-phase protein found to be activated by the host immune system during stress conditions [40]. Animals undergoing an external or internal challenge to their state of health mount a vigorous response, including the acute-phase response, to limit the harmful effects of the stimulus. Terech-Majewska [13] did not notice statistically significant changes in the level of ceruloplasmin in serum after the use of formalin-inactivated cells from *A. salmonicida*, *A. sobria*, *A. hydrophila*, and *P. fluorescens* by immersion, injection, or feed in multivalent vaccine. In the case of our results for ceruloplasmin, its level decreased in the groups vaccinated with feed and injection in the first week after vaccination. The evaluation of ceruloplasmin activity also serves as an indicator of liver function. Its increase is an indicator of liver toxicity. In the first collection, a decrease in the level of activity was found in the groups where the vaccine was administered with the feed and injection. This confirms the differential effect of vaccination methods on liver function. Ceruloplasmin is a circulating protein derived from the liver that is important in the release of iron from certain tissues. Although it is predominant in serum copper in many species, the absence of ceruloplasmin does not lead to disturbance of copper homeostasis; rather, it has a preferential effect on iron metabolism [41]. After 14 days in our study, it did not differ from the control in any experimental group. This confirms that the changes were temporary.

Preventive programs based on commercial vaccines or autovaccinations are nowadays gradually displacing programs based on the use of therapeutic agents. Protection can based on commercial preparations or autovaccines. The economic aspects of immunoprophylaxis are easy to calculate by the possibility of reducing losses. Vaccinations can be preparatory to a new environment, facilitating adaptation to new conditions. Pikeperch make such a transition, as their initial period of controlled rearing is carried out in closed RAS systems, while at a later stage they are moved to ponds or used for restocking in the natural environment. The killed forms of pathogens that are appropriate for the epizootic situation on a given farm can be placed in the autovaccine composition [20]. The advantage of an autovaccine is the ability to use it on farms without the need to register it, because there are no such legal requirements for autovaccines. This gives an additional opportunity to protect fish health, especially new species in aquaculture and fish for which no commercial vaccines exist.

## 5. Conclusions

When choosing a vaccination method, one should take into account not only the immunoprotection that may be granted but also the harm that may be caused to fish. In the light of the research results obtained in the rearing of zander, vaccination can be used with the tested methods, integrating them into targeted preventive programs. The intraperitoneal vaccination route against *Aeromonas salmonicida* in juvenile pikeperch shows the greatest effectiveness, taking into account the limitations of mortality after experimental infection. The use of an autovaccine, regardless of the route of administration, does not affect morphological or histological indicators of the liver.

## Figures and Tables

**Table 1:** Rearing indicators of pikeperch after vaccination against *A. salmonicida* by various routes (mean value ± SD, *p* ≤ 0.05).

Parameters	Fish Groups			
	C	I	B	F
BL (cm)	-	-	-	-
d0	14.11 (±0.54)	14.09 (±0.58)	14.11 (±0.58)	14.10 (±0.57)
**d28**	**15.98** **(±0.75)** ^**a,b**^	**15.43** **(±1.09)** ^**a**^	**16.21** **(±0.77)** ^**b**^	**16.00** **(±0.70)** ^**a,b**^
**d56**	**17.57** **(±1.00)** ^**a,b**^	**16.93** **(±1.65)** ^**a**^	**17.86** **(±1.07)** ^**a,b**^	**17.90** **(±0.85)** ^**b**^
BW (g)	-	-	-	-
d0	34.58 (±3.97)	34.38 (±3.77)	34.27 (±3.93)	34.22 (±3.96)
**d28**	**52.16** **(±8.84)** ^**a,b**^	**44.83** **(±11.31)** ^**a**^	**52.91** **(±7.62)** ^**b**^	**50.85** **(±6.59)** ^**a,b**^
d56	67.09 (±14.65)	59.57 (±18.7)	68.70 (±13.70)	69.81 (±10.87)
DGR (g/day)	-	-	-	-
d0–d28	0.63 (±0.04)	0.38 (±0.05)	0.67 (±0.06)	0.59 (±0.07)
d29–d56	0.55 (±0.06)	0.56 (±0.12)	0.58 (±0.13)	0.70 (±0.06)
d0–d56	0.59 (±0.04)	0.47 (±0.09)	0.63 (±0.09)	0.65 (±0.05)
SGR (%/day)	-	-	-	-
d0–d28	1.47 (±0.09)	0.96 (±0.12)	1.55 (±0.11)	1.41 (±0.14)
d29–d56	0.93 (±0.10)	1.06 (±0.17)	0.96 (±0.15)	1.17 (±0.07)
d0–d56	1.20 (±0.07)	1.01 (±0.14)	1.26 (±0.13)	1.30 (±0.08)
CF (-)	-	-	-	-
d0	1.23 (±0.05)	1.23 (±0.06)	1.22 (±0.06)	1.23 (±0.05)
d28	1.27 (±0.08)	1.20 (±0.12)	1.24 (±0.05)	1.24 (±0.05)
d56	1.22 (±0.08)	1.18 (±0.12)	1.19 (±0.06)	1.21 (±0.06)
FCR (-)	-	-	-	-
d0–d28	0.86 (±0.07)	1.66 (±0.34)	0.88 (±0.07)	1.00 (±0.07)
d29–d56	1.40 (±0.11)	1.28 (±0.23)	1.37 (±0.24)	1.15 (±0.09)
d0–d56	1.04 (±0.05)	1.45 (±0.25)	1.05 (±0.08)	1.07 (±0.08)
Survival (%)	-	-	-	-
d0–d28	100.0 (±0.00)	79.0 (±7.22)	100.0 (±0.00)	100.0 (±0.00)
d29–d56	100.0 (±0.00)	100.0 (±0.0)	100.0 (±0.00)	100.0 (±0.00)
d0–d56	100.0 (±0.00)	79.0 (±7.22)	100.0 (±0.00)	100.0 (±0.00)

Different letters in the table point statistical significance.

**Table 2:** Hematological indexes of pikeperch after vaccination against *A. salmonicida* by various routes (mean value ± SD, *n* = 8, *p* ≤ 0.05).

Parameter	Units	Fish Groups			
		C	I	B	F
**WBC**	10^3^/µL	69.45 ± 13.62	63.00 ± 7.01	53.29 ± 5.63	52.24 ± 8.56
RBC	10^6^/µL	1.58 ± 0.25	1.57 ± 0.13	1.43 ± 0.09	1.44 ± 0.09
HGB	g/L	25.54 ± 3.27	24.78 ± 1.87	23.26 ± 1.40	23.35 ± 1.89
HCT	%	27.93 ± 4.09	27.12 ± 2.40	26.16 ± 1.46	25.73 ± 1.78
**MCV**	**fL**	**131.65 ± 4.47** ^**a,b**^	**128.01 ± 4.55** ^**a**^	**136.00 ± 2.91** ^**b**^	**132.74 ± 1.55** ^**a,b**^
MCH	pg	26.61 ± 1.26	25.83 ± 0.96	26.68 ± 1.06	26.53 ± 0.56
MCHC	g/L	202.50 ± 6.97	202.00 ± 4.84	196.38 ± 4.63	200.29 ± 3.40
PLT	10^3^/µL	21.29 ± 3.25	18.00 ± 6.06	17.38 ± 4.93	16.29 ± 3.40

Different letters in the table point statistical significance.

**Table 3:** Biochemical parameters of pikeperch after vaccination against *A. salmonicida* by various routes (mean value ± SD, *n* = 8, *p* ≤ 0.05).

Parameter	Units	Fish Groups			
		C	I	B	F
**CREA**	mg/dL	0.24 ± 0.08	0.23 ± 0.12	0.27 ± 0.09	0.31 ± 0.16
TP	g/dL	4.43 ± 0.33	4.28 ± 0.36	4.24 ± 0.17	4.51 ± 0.24
**BIL-T**	**mg/dL**	**0.12 ± 0.06** ^**b**^	**0.09 ± 0.02** ^**a,b**^	**0.06 ± 0.03** ^**a**^	**0.07 ± 0.02** ^**a,b**^
ALT	U/L	26.88 ± 8.15	35.38 ± 21.59	23.13 ± 9.67	24.13 ± 14.17
ALP	U/L	162.38 ± 46.90	183.38 ± 69.19	138.00 ± 32.86	126.38 ± 17.54
Ca	mg/dL	11.51 ± 0.41	11.09 ± 0.58	11.33 ± 0.40	11.63 ± 0.43
ALB	g/dL	1.72 ± 0.16	1.65 ± 0.16	1.71 ± 0.06	1.76 ± 0.09
GLB	g/dL	2.71 ± 0.18	2.63 ± 0.23	2.52 ± 0.15	2.75 ± 0.1
AST	U/L	92.88 ± 25.79	112.50 ± 41.73	85.50 ± 48.54	115.13 ± 81.23
GLU	mg/dL	98.50 ± 16.31	116.63 ± 35.26	88.63 ± 15.68	92.50 ± 14.64
Cl	mmol/L	327.93 ± 21.38	312.21 ± 22.96	299.08 ± 26.77	321.78 ± 17.52
Mg	mg/dL	3.15 ± 0.16	3.03 ± 0.28	3.08 ± 0.21	3.25 ± 0.09
LACT	mg/dL	2.85 ± 0.73	2.71 ± 0.99	2.30 ± 0.73	2.78 ± 0.84
NH_3_	µg/dL	58.59 ± 22.66	50.94 ± 7.90	46.23 ± 7.79	59.56 ± 28.57

Different letters in the table point statistical significance.

**Table 4:** Morphological and histological indicators of pikeperch liver (mean value ± SD, *n* = 8, *p* ≤ 0.05).

Parameter	Fish Groups			
	C	I	B	F
HIS (%)	1.49 ± 0.37	1.69 ± 0.27	1.38 ± 0.35	1.42 ± 0.29
HD (μm)	14.70 ± 1.53	14.79 ± 1.43	13.88 ± 0.88	14.25 ± 1.11
	(13.48−17.20)	(13.02–17.42)	(12.28−14.95)	(12.09−15.53)
ND (μm)	3.65 ± 0.17	3.59 ± 0.17	3.60 ± 0.09	3.69 ± 0.01
	(3.38−3.94)	(3.37–3.79)	(3.46–3.76)	(3.59–3.86)
INCP	0.25 ± 0.02	0.25 ± 0.03	0.26 ± 0.02	0.26 ± 0.02
	(0.21–0.29)	(0.20–0.29)	(0.23–0.31)	(0.24–0.31)
